# The Association Between Prenatal Infection and Adolescent Behavior: Investigating Multiple Prenatal, Perinatal, and Childhood Second Hits

**DOI:** 10.1016/j.jaac.2023.06.009

**Published:** 2023-06-23

**Authors:** Anna Suleri, Tonya White, Elisabet Blok, Charlotte A.M. Cecil, Irwin Reiss, Vincent W.V. Jaddoe, F.A.J. Gigase, Manon H.J. Hillegers, Lot de Witte, Veerle Bergink, Anna-Sophie Rommel

**Affiliations:** Erasmus University Medical Center, Rotterdam, the Netherlands; Erasmus University Medical Center, Rotterdam, the Netherlands; Erasmus University Medical Center, Rotterdam, the Netherlands; Erasmus University Medical Center, Rotterdam, the Netherlands; Erasmus University Medical Center, Rotterdam, the Netherlands; Erasmus University Medical Center, Rotterdam, the Netherlands; Erasmus University Medical Center, Rotterdam, the Netherlands; Erasmus University Medical Center, Rotterdam, the Netherlands; Icahn School of Medicine at Mount Sinai, New York; Erasmus University Medical Center, Rotterdam, the Netherlands, Icahn School of Medicine at Mount Sinai, New York; Icahn School of Medicine at Mount Sinai, New York

**Keywords:** maternal immune activation, neurodevelopment, maternal health, pregnancy, child health

## Abstract

**Objective:**

Exposure to infections during pregnancy may be a potential risk factor for later psychopathology, but large-scale epidemiological studies investigating associations between prenatal infection and long-term offspring behavioral problems in the general population are scarce. In our study, we aimed to investigate the following: (1) the association between prenatal infection and adolescent behavior, (2) putative underlying pathways (mediation), and (3) “second hits” interacting with prenatal infection to increase the risk of adolescent behavior problems (moderation).

**Method:**

Our study was embedded in a prospective Dutch pregnancy cohort (Generation R; n = 2,213 mother–child dyads). We constructed a comprehensive prenatal infection score comprising common infections for each trimester of pregnancy. At age 13 to 16 years, we assessed total, internalizing, and externalizing problems, and autistic traits using the Child Behavioral Checklist and the Social Responsiveness Scale, respectively. We investigated maternal lifestyle and nutrition, perinatal factors (placental health and delivery outcomes), and child health (lifestyle, traumatic events, infections) as mediators and moderators.

**Results:**

We observed associations of prenatal infection with adolescent total behavioral, internalizing, and externalizing problems. The association between prenatal infection and internalizing problems was moderated by higher levels of maternal psychopathology, alcohol and tobacco use, and a higher number of traumatic childhood events. We found no association between prenatal infection and autistic traits. Yet, children exposed to prenatal infections and maternal substance use, and/or traumatic childhood events, had a higher risk of autistic traits in adolescence.

**Conclusion:**

Prenatal infection may be a risk factor for later psychiatric problems as well as a disease primer making individuals susceptible to other hits later in life.

**Study preregistration information:**

Prenatal maternal infection and adverse neurodevelopment: a structural equation modelling approach to downstream environmental hits; https://osf.io/cp85a; cp85a.

Exposure to infections during pregnancy is increasingly recognized as a potential risk factor for psychopathology and neurodevelopmental disorders in offspring, including increased risk for autism spectrum disorder (ASD), depression, and attention-deficit/hyperactivity disorder (ADHD).^1^ Infectious stimuli can cross the placenta directly or assert their effects via dysregulation of the maternal immune system.^2^ However, several recent reviews and meta-analyses dedicated to this topic, including the most recent and largest meta-analysis,^3^ noted the limited quantity and quality of studies on the association between prenatal infection and behavioral problems. In brief, this review noted that despite the growing body of literature on prenatal infection and psychopathology, most studies are small and do not adjust for important confounders. Thus, the reproducibility of the results is affected. Moreover, existing studies focus on clinical cases of infection, for example by studying hospitalized mothers with respiratory infections such as influenza or perinatal infections such as chorioamnionitis. Moreover, prior studies have tended to focus on clinical diagnoses of psychopathology (eg, ASD, ADHD). The generalizability to a broader range of infections (concerning both severity and type) and behavioral problems along the continuum is therefore limited.^1,3–5^ To date, large-scale epidemiological studies investigating the long-term association between prenatal infection and offspring behavioral problems in the general population are scarce.

In humans, it is challenging to study the potential pathway underlying the association between infections during pregnancy and later-life offspring behavioral problems because this pathway is highly complex, with several environmental factors affecting the association over the course of a lifetime.^2^ As such, prior studies have focused on the total effect of prenatal infection on behavioral problems. Different mediators, namely, causal factors on the pathway from prenatal infection to adolescent behavior problems, may play an important role. For example, because placental histopathology has been linked to both prenatal infection^6^ and ASD,^7^ placental abnormalities may be an important mediator underlying the association. Similarly, delivery outcomes such as preterm birth could be causalities on the pathway from prenatal infection to adverse neurodevelopment.^8^

In addition to a direct association between prenatal infection and behavioral problems, the “second-hit hypothesis” suggests that prenatal infections may act only as disease primer. As such, prenatal infections may not induce behavioral problems by themselves but increase an individual’s susceptibility to downstream stressors (“second hits”).^2^ Normally, when a “hit” occurs, the individual’s inflammatory response protects against such stressors. Yet, if the fetal immune system was exposed to prenatal infections and imprinted atypically, the individual’s lifelong susceptibility to disease may increase.^2^ Maternal psychopathology, deficient maternal nutrition, high body mass index (BMI), and maladaptive lifestyle factors (eg, smoking) during pregnancy may be “second hits” of interest (hereafter defined as moderating factors potentially interacting with prenatal infection to increase the risk of adolescent behavior problems).^2,9,10^

Here, we aimed to investigate the association between prenatal exposure to common infections and a range of adolescent behavioral problems at age 13–16 years in a large population-based cohort. We considered the effects of prenatal infections during the whole pregnancy, as well as in each trimester separately. We further aimed to examine putative pathways underlying the association between prenatal infection and adolescent behavior by studying potential perinatal mediators (eg, placenta growth and blood flow, birthweight, and gestational age at birth). Finally, we explored the possibility of “second hits,” namely, moderating factors interacting with prenatal infection to increase the risk of adolescent behavior problems. We examined maternal (eg, nutrition, lifestyle, pregnancy health) and childhood (eg, traumatic events, lifestyle, infections, and BMI) moderating factors.

## METHOD

### Preregistration

We preregistered our study prior to any analyses (https://osf.io/cp85a) (Supplement 1 and Tables S1-S3, available online).

### Study Selection and Participants

This study was embedded in the Generation R Study, a large prospective population-based cohort investigating child development from pregnancy onwards.^11^ Pregnant individuals (N = 9,778) living in Rotterdam, the Netherlands, were recruited between April 2002 and January 2006. Prenatally, mothers were followed up during each trimester (~70% enrolled in the first trimester). Postnatally, mothers and their children were assessed in multiple follow-up waves at mean ages of 6, 10, 14, and currently at age 17 years. The Medical Ethics Committee of the Erasmus Medical Centre approved all study procedures. Parents and their children provided written informed consent.

**FIGURE 1:**
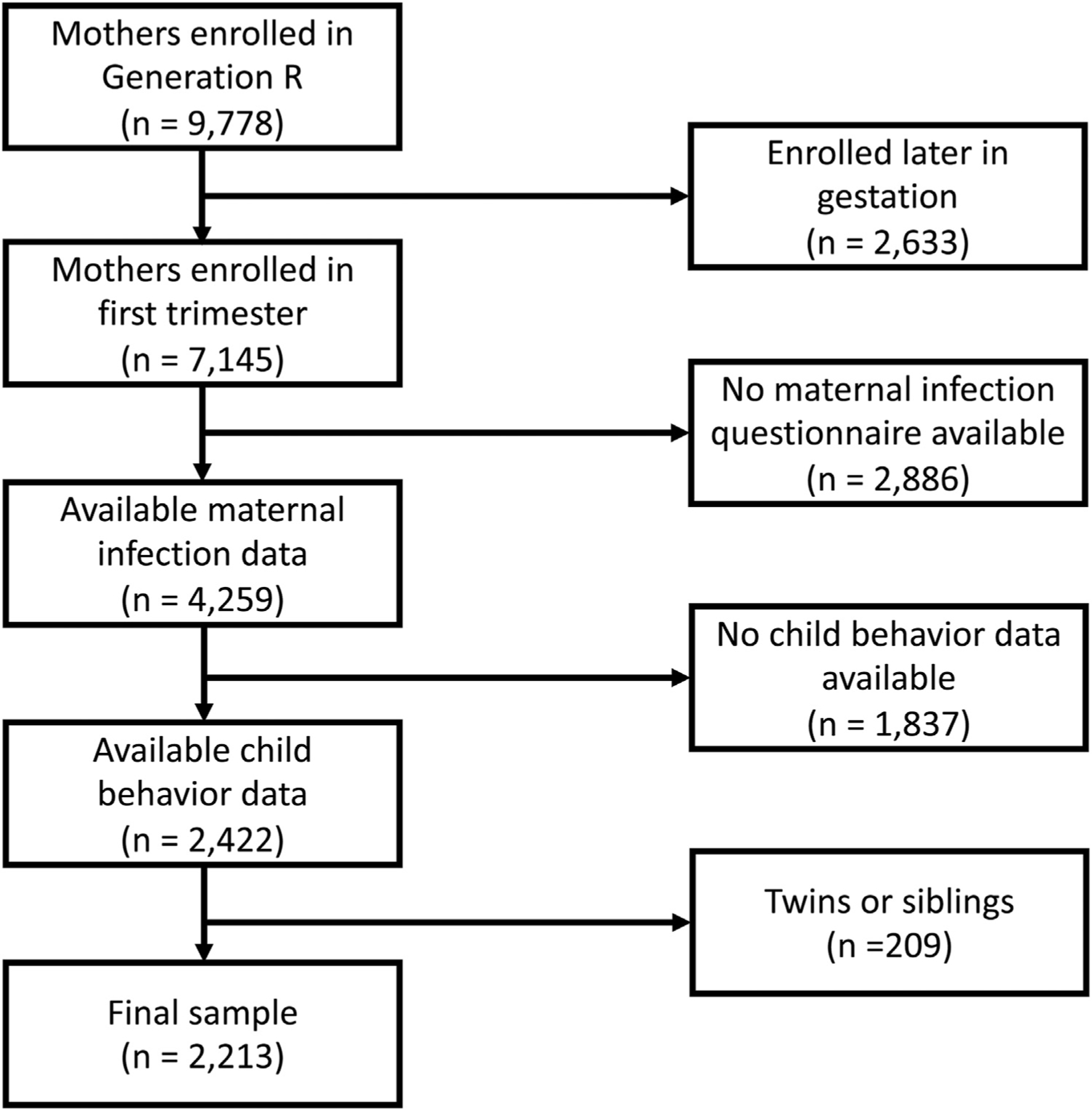
Flow Chart of Study Population Note: This figure shows the flowchart of the study population (application of the inclusion and exclusion criteria).

To be included in our study, mothers had to be enrolled in their first trimester, and information on prenatal infection during each trimester as well as information on the parent-reported behavior outcomes at the 13 to 16 years assessment wave had to be available. For siblings and twins, 1 sibling/twin was excluded at random. After applying these criteria, 2,213 mother–child pairs were included in our study (Figure 1).

### Exposure: Prenatal Infection Assessment

To define prenatal infections, we used complete cases of questionnaire data collected at 3 time points during pregnancy, specifically at the end of trimesters 1, 2, and 3. Women were asked to report on the following infection items: upper respiratory infections (pharyngitis, rhinitis, sinusitis, ear infection), lower respiratory infections (pneumonia, bronchitis), gastrointestinal infections (diarrhea, enteritis), cystitis/pyelitis, dermatitis (boils, erysipelas), eye infections, herpes zoster, flu, sexually transmitted diseases (STD), and a period of fever (>38°C/100.4°F) within the past 3 months (first and third trimester) or 2 months (second trimester) (Supplement 2, Figure S1, available online).

We constructed 4 prenatal infection sum scores: 1 score for each trimester (trimester-based), and 1 score for the whole pregnancy. For the trimester-based scores, each confirmation of a condition within that trimester (“yes”) was scored as 1 point. A “no” response was rated with 0 points. A maximum sum score of 10 points could be derived per trimester (maximum 30 points for the whole pregnancy). Fever was additionally used as a separate severity marker. We constructed a binary fever variable (yes/no) for each trimester and a sum score for the whole pregnancy (maximum 3 points per pregnancy).

The Generation R cohort has data on prenatal C-reactive protein (CRP) levels available at a single time-point (mean 12 weeks of gestation), although not at timing of infection. Given the short half-life (~12 hours) of CRP^12^ and the phenomenon of healthy volunteer bias (ie, infected participants are less likely to attend a research visit), we were unable to use CRP levels in our prenatal infection score (Supplement 3, Figure S3, displays the correlation matrix between infections and CRP, available online).

### Outcome: Adolescent Behavior Problems

#### Child Behavioral Checklist.

To examine internalizing, externalizing and total behavioral problems, we used parental ratings on the Child Behavioral Checklist (CBCL) at age 13 to 16 years.^13^ Parents reported on the adolescents’ behavior over the past 6 months. Eight empirically based scales were derived from 112 items: anxious/depressed, withdrawn/depressed, somatic complaints, social problems, thought problems, attention problems, rule-breaking behavior, and aggressive behavior. Those scales were then combined into externalizing problems (rule-breaking behavior, aggressive behavior), and internalizing problems (anxious/depressed, withdrawn/depressed, somatic complaints). The total behavioral problems score comprises externalizing and internalizing problems, and additionally includes social problems, thought problems, and attention problems. Higher scores indicate higher levels of behavioral problems (Supplement 2, Figure S2, available online).

#### Social Responsiveness Scale.

We measured autistic traits in adolescents at age 13 to 16 years, using the abbreviated parent-reported Social Responsiveness Scale (SRS; 18-items).^14^ The abbreviated SRS is a validated questionnaire assessing interpersonal behavior, communication, and repetitive/stereotypic behavior in adolescents. Higher scores indicate higher levels of autistic traits (Supplement 2, Figure S2, available online).

### Mediators

Levels of placental growth factor (PIGF) were measured in the first 18 weeks of gestation in maternal venous blood samples. The arteria umbilicalis pulsatility index and arteria uterine resistance index were obtained by ultrasound examinations in the second trimester. The 5-minute Apgar score, umbilical cord blood pH, placental weight at birth, gestational age at birth, and birthweight were obtained from hospital registries (standardized delivery registrations of midwives and obstetricians). Supplements 4 and 5, available online, provide more information on these mediators.

### Moderators

Pre-pregnancy BMI was calculated based on height and weight before pregnancy from the enrollment questionnaire. The enrollment questionnaire also included questions regarding psychoactive substance use, tobacco use, and alcohol consumption during pregnancy. Maternal psychopathology was assessed at enrollment using the Global Severity Index from the Brief Symptom Inventory.^15^ The diet quality score was calculated based on the food frequency questionnaire administered during the first 18 weeks of gestation.^16^ Iron and vitamin D levels were measured in the first 18 weeks of gestation in maternal venous blood samples. Information about breastfeeding was obtained from questionnaires 12 months after birth. Childhood BMI was measured at the 9 to 12 years research visit. A sum score for childhood infection was constructed based on questionnaire data asking about antibiotic use at 2 and 6 months and at 1, 2, 3, 4, 5, and 9 years of age (for details on score construction, see Table S4). Childhood trauma was assessed using cumulative scores of postnatal life events and postnatal direct victimization (https://github.com/SereDef/cumulative-ELS-score).^17^ Adolescent alcohol and tobacco use were assessed in self-reported questionnaires at 13 to 16 years. Supplement 6, available online, provides more information.

### Covariates

**FIGURE 2:**
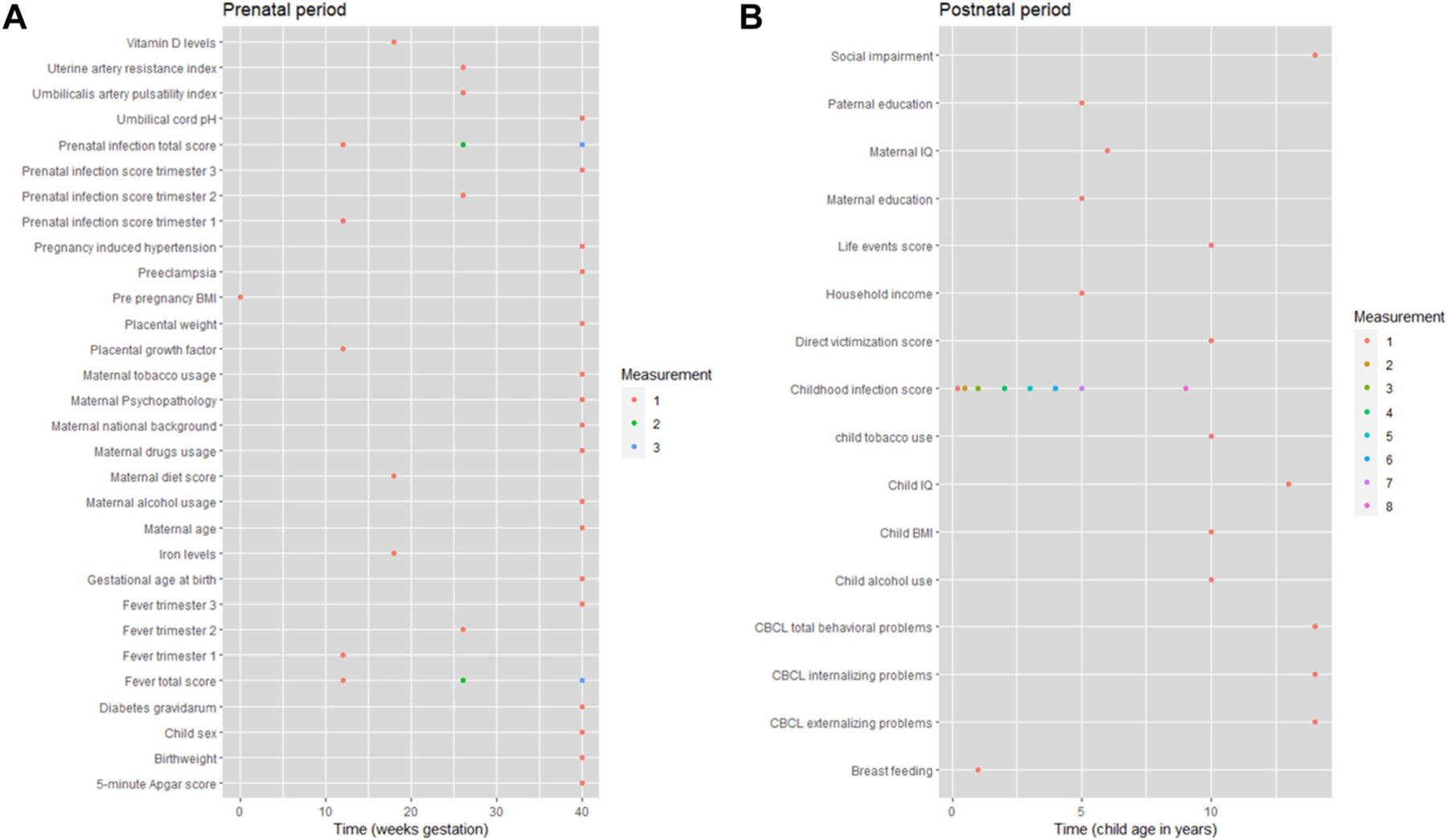
Overview of Included Variables Note: This plot, in 2 panels, shows when each variable (exposure, outcomes, mediators, moderators, and covariates) was measured. BMI = body mass index; IQ = intelligence quotient; pH = potential of hydrogen.

Child age was established from the date of birth and the date of questionnaire completion. Hospital registries provided information on child sex. Maternal age and national background (“Dutch” or “non-Dutch”) were established via the enrollment questionnaire. Household income at enrollment (<€2,200 per month or >€2,200 per month), parental education (primary [no education or primary school], intermediate [secondary school or lower vocational training] and high [higher vocational training or university]), and maternal IQ (Raven’s Advanced Progressive Matrices Test set I^18^) were assessed when the child was 5 to 8 years of age. Child IQ was assessed using the vocabulary, matrix reasoning, digit span, and coding subscales of the Wechsler Intelligence Scale for Children–Fifth Edition at age 13 to 16 years.^19^ Figure 2 shows an overview of the measurement time of each exposure, outcome, mediator, moderator, and covariate variable.

### Statistical Analyses

We performed a non-response analysis to explore subsample selection biases. We compared the demographical variables of study participants to those of the whole Generation R sample. We used χ^2^ tests for categorical variables and unpaired *t* tests for continuous variables. We square root transformed the CBCL and SRS scores to satisfy the normality assumption. To enable direct comparison between variables, we standardized all variables to *z* scores.

#### Direct Association.

We used linear regressions to investigate the associations between prenatal infection and behavioral problems. As exposure, we first used the total infection sum score for the whole pregnancy. We ran separate analyses for the 4 outcome variables: CBCL total behavioral, internalizing and externalizing problems, and SRS total score. To investigate potential sensitive periods, we subsequently examined associations between the 3 trimester-based infection sum scores and the 4 behavioral outcomes in individual models.

#### Mediation.

We applied separate mediation analyses for each behavioral outcome (CBCL total behavioral, internalizing, and externalizing problems; and SRS total score). For each analysis, we included the following mediators into the same model: arteria umbilicalis pulsatility index, arteria uterine resistance index, placental growth factor, placental weight, birthweight, and gestational age at birth. We used bootstrapping (n = 1,000) to obtain 95% confidance intervals and *p* values for each indirect path in the mediation model.

#### Moderation.

We conducted separate moderation analyses for various maternal and child moderators. We added interaction terms between prenatal infection and the following maternal variables: psychopathology, dietary food score, iron levels, vitamin D levels, pre-pregnancy BMI, substance use, alcohol use, tobacco use; and interaction terms between prenatal infection and the following child variables: BMI, tobacco use, alcohol use, breastfeeding, infections, postnatal life events score, and postnatal direct victimization score.

#### Sensitivity Analyses.

To investigate the effect of infection severity, we repeated the linear regressions for the direct associations using “fever” instead of “prenatal infection” as exposure. To investigate the role of child sex, we included an interaction term between prenatal infection and child sex. We repeated the analyses for all infection types separately to investigate whether there was an infection-specific effect.

#### Additional Information.

All models were adjusted for the following covariates: maternal age,^20^ maternal education,^21^ paternal education,^22^ household income,^23^ maternal national background,^24^ maternal IQ,^25^ child IQ,^26^ child sex,^27^ and child age.^28^ The rationale behind these covariates was that these may be associated with prenatal infection and/or behavioral problems in adolescence but are not on the causal pathway underlying the association. Figure S4 and Supplement 4, available online, show an overview of all aims, variables, and analyses. The *p* values presented throughout the article are false discovery rate–Benjamini Hochberg corrected, with a *p*_corrected_ value ≤.05 considered significant. All analyses were conducted in R (version 3.6.1). Missing moderator (maximum 30%) and covariate (maximum 15%) data were imputed in R with multiple imputation using chained equations (30 iterations and 8 imputations; “mice” package). For missing mediator data, we applied the default method in the “lavaan” package (full information maximum likelihood), where these models were conducted.

## RESULTS

### Participant Demographics

A total of 2,213 mother–child pairs were included in our study. Table 1 contains descriptive information. Supplement 7 and Table S5, available online, contain the frequencies of the moderators and mediators, respectively. Supplement 8, available online, describes the non-response analysis.

### Direction Association

After adjusting for multiple testing, we found direct associations between the total infection sum score for the whole pregnancy and CBCL total behavioral problems (b = 0.106, 95% CI = 0.065–0.148, *p*_corrected_ < .001), CBCL internalizing problems (b = 0.111, 95% CI = 0.069–0.152, *p*_corrected_ < .001), and CBCL externalizing problems (b = 0.057, 95% CI = 0.015–0.099, *p*_corrected_ < .050) (Table 2, and Supplement 9 and Figure S5, available online). We found no direct association between the total infection sum score for the whole pregnancy and SRS total score (b = 0.021, 95% CI = –0.020 to 0.062, *p*_corrected_ = .623). We observed similar associations for the trimester-based infection sum scores (Table 2).

### Mediation

None of the placental health variables (arteria umbilicalis pulsatility index, arteria uterine resistance index, placental growth factor, and placental weight) or delivery outcomes (birthweight, gestational age at birth, 5-minute Apgar score, and umbilical cord blood pH) mediated the associations between prenatal infection and adolescent behavior (Supplement 10 and Table S6, available online).

### Moderation

The association between prenatal infection during pregnancy and CBCL total behavioral problems was moderated by higher levels of maternal psychopathology (b = 0.067, 95% CI = 0.033–0.101, *p*_corrected_ < .010), maternal alcohol use (b = 0.153, 95% CI = 0.086–0.218, *p*_corrected_ < .001), maternal tobacco use (b = 0.145, 95% CI = 0.034–0.256, *p*_corrected_ < .050), child postnatal life events (b = 0.058, 95% CI = 0.019–0.096, *p*_corrected_ < .050), and child postnatal direct victimization (b = 0.075, 95% CI = 0.035–0.114, *p*_corrected_ < .010) (Table 3).

The association between prenatal infection during pregnancy and CBCL internalizing problems was moderated by higher levels of maternal psychopathology (b = 0.062, 95% CI = 0.028–0.095, *p*_corrected_ < .010), maternal substance use (b = 0.215, 95% CI = 0.064–0.366, *p*_corrected_ < .050), maternal alcohol use (b = 0.127, 95% CI = 0.062–0.193, *p*_corrected_ < .010), maternal tobacco use (b = 0.183, 95% CI = 0.071–0.293, *p*_corrected_ < .010), child alcohol use (b = 0.270, 95% CI = 0.068–0.472, *p*_corrected_ < .50), child postnatal life events (b = 0.057, 95% CI = 0.019–0.096, *p*_corrected_ < .050), and child postnatal direct victimization (b = 0.074, 95% CI = 0.035–0.114, *p*_corrected_ < .010). The association between prenatal infection during pregnancy and CBCL externalizing problems was not moderated by any of the investigated factors.

The association between prenatal infection during pregnancy and SRS total score was moderated by higher levels of maternal substance use (b = 0.221, 95% CI = 0.073–0.369, *p*_corrected_ < .050), child postnatal life events (b = 0.013, 95% CI = 0.013–0.090, *p*_corrected_ < .050), and child postnatal direct victimization (b = 0.054, 95% CI = 0.015–0.094, *p*_corrected_ < .050).

### Sensitivity Analyses

Our sensitivity analyses showed no effect of fever (Supplement 11 and Table S7, available online) and no moderating effect of child sex (Table S8, available online). The individual results per infection type for each behavior outcome can be found in Table S9 (available online).

## DISCUSSION

In this large population-based cohort, we examined the relationship between prenatal infection and behavioral problems at age 13 to 16 years. We further examined the putative mechanism underlying this association (mediation) and the presence of “second hits” (moderation) that interact with prenatal infections to increase the risk of behavioral problems. We highlight 3 key findings. First, we observed associations between prenatal infection and total behavioral, internalizing, and externalizing problems. We found no association between prenatal infection and autistic traits. We observed similar associations for trimester-based scores. Second, none of the investigated perinatal variables were on the pathway from prenatal infection to adolescent behavior. Third, we observed that maternal psychopathology, maternal alcohol use, maternal tobacco use, child postnatal life events, and child postnatal direct victimization interacted with prenatal infections to strengthen the associations with adolescent total behavioral and internalizing problems. Maternal substance use and child alcohol use further moderated the association between prenatal infection and internalizing problems. Maternal substance use, child postnatal life events and child postnatal direct victimization interacted with prenatal infection to increase the risk of adolescent autistic traits.

Our finding of direct associations between prenatal infection and adolescent total behavioral, internalizing, and externalizing problems are in line with previous research.^29^ The relationship between prenatal infection and child internalizing behavior, particularly depressive and anxious symptomology, has been well documented across the life-span.^29^ The association between prenatal infection and externalizing symptoms is less well established. Although several studies have indicated that prenatal infection is associated with reduced impulse control in toddlers^30^ and a higher risk of ADHD in the child,^31^ other studies have not observed an association on externalizing problems, suggesting that the association may be better explained by unmeasured familial confounding^32^. However, heterogeneity across studies is high.^33^ Our study adds to the existing evidence, suggesting that the previously reported associations between severe infections and clinical diagnoses of psychiatric conditions also extend to common infections and continuously measured internalizing and externalizing behavior. These effects persisted after correction for multiple confounders and multiple testing—which were novel features of our study—increasing the robustness of the results.

Infections during pregnancy may affect the presence of internalizing and externalizing problems in children through various pathways.^1,2^ Vertical transmission, whereby the pathogen directly crosses the placenta and affects fetal development, is 1 potential pathway. Another possible pathway is the maternal immune system’s inflammatory response to the infection, rather than the pathogen itself. This response could cause immune cells to cross the placenta, to activate fetal immune cells, and consequently to affect fetal neurodevelopment.

Moreover, although various environmental risk factors have been linked to prenatal infections or adolescent behavior, our study is among the first to examine the joint role of multiple mediators in the relationship between prenatal infection and adolescent behavior. Because we found no mediating effects of placental health and birth outcomes, our findings suggest that perinatal outcomes do not to lie on the pathway from prenatal infection to adolescent behavioral problems.

In line with the “second-hits hypothesis,” we investigated various moderating factors. We observed that maternal lifestyle and traumatic childhood events strengthened the association between prenatal infection and adolescent behavioral problems. Maternal psychopathology may assert its effect via suboptimal parenting practices and/or genetic vulnerability.^34^ Similarly, a maladaptive lifestyle may have an exacerbating effect on the association between prenatal infection and adolescent behavior. Lifestyle factors such as alcohol and tobacco use might interact with the immune system.^2^ Postnatal traumatic events may lead to a chronic stress response in the child, which could interact with the altered immune response caused by prenatal exposure to infection.^35^ The relatively high heritability of externalizing symptoms compared to internalizing problems (eg, h^2^ = 74% for conduct disorder,^36^ h^2^ = 37% for depression^37^) may be the reason why none of the environmental factors investigated here interacted with prenatal infection to increase the risk for externalizing problems.

Although earlier work investigating prenatal infection and fever reported a positive association with ASD,^38^ we found no association between prenatal infection and autistic traits. To complement previous research on clinical ASD, we studied autistic traits in the general population, and adjusted for several important confounders and multiple testing to reduce the risk of false-positive results. In line with our findings, a meta-analysis showed no effect of prenatal infections on autism risk.^39^ Moreover, a recent large-scale Swedish register-based study concluded that the association between prenatal infection and autism may not reflect a causal relationship, but may be better explained by shared familial factors (e.g., genetic variation or shared environment).^40^ Although we did not observe a direct association between prenatal infection and autistic traits, prenatal maternal substance use and childhood traumatic experiences interacted with prenatal infection to increase the risk of autistic traits. Thus, prenatal infection may be a disease primer for autistic traits, making individuals susceptible to other hits later in life.

The strengths of our study include the large population-based cohort, the broad range of infections, the long follow-up, and the inclusion of moderators, mediators, and relevant covariates. Moreover, we applied multiple testing corrections to minimize the probability of false-positive results. The study also has limitations. One limitation was that prenatal infections were recorded retrospectively, which may have introduced some recall bias. However, we attempted to minimize this bias by asking participants to recall infections after each trimester, which resulted in a recall period of approximately 2 to 3 months. In addition, self-report questionnaires rather than blood serum biomarkers were used. Self-report may be subject to recall bias. Yet, self-report questionnaires may also be advantageous, because they are not affected by healthy volunteer bias as they could be filled in during any point in the trimester at home, whereas infected participants are less likely to attend research visits where the blood withdrawal measurement occurs. Nonetheless, future research would benefit from serological evidence for bacterial and viral infections in each trimester of pregnancy to ascertain the medical diagnosis. Another limitation was that we were unable to investigate systemic immune responses because we did not have blood samples taken at the time of infection. This could have limited our understanding of the mechanisms underlying the observed associations. Furthermore, when using fever as a marker of infection severity, we found no associations with adolescent behavior, which may have been due to inadequate statistical power. Our study was designed primarily to detect potential long-term effects of exposure to common infections on child behavioral problems, given that most prior literature has focused on the more severe infections.

In conclusion, the results of this large population-based cohort show that prenatal infection may increase the risk for some adolescent behavioral problems. Maternal psychopathology, a maladaptive lifestyle during pregnancy, child alcohol use, and traumatic childhood events may interact with prenatal infections to further increase the risk for behavior problems. Although it may be difficult for pregnant individuals to prevent exposure to common infections, public health measures may be used to address the need to increase resilience and resistance to infections and to prevent the spread of diseases. Public health measures aimed at the pregnant individual may comprise promoting healthy lifestyle choices such as getting enough rest and exercise, maintaining a balanced diet, and staying up to date with vaccinations during pregnancy. In addition, community-level public health efforts implemented to reduce the transmission of infections may include improving ventilation and providing education and resources on hand hygiene, mask wearing and social distancing in public settings.

## Supplementary Material

supplementary material

## Figures and Tables

**TABLE 1:** Descriptive Characteristics of Participants

Mothers	
General characteristics	
Age at enrollment. mean ± SD	31.3 ± 4.3
Pre-pregnancy BMI, mean ± SD	23.2 ± 4.0
Maternal IQ, mean ± SD	100.2 ± 13.3
National background	
Dutch, n (%)	1,549 (70.0)
Non-Dutch, n (%)	656 (29.6)
Maternal education	
High, n (%)	1,400 (63.3)
Intermediate, n (%)	631 (28.5)
Low, n (%)	15 (0.7)
Household income	
< €2000 (%)	197 (8.9)
> €2000 (%)	1,747 (78.9)
Exposure	
Total pregnancy prenatal infection sum score, mean ± SD	2.9 ± 2.1
Children	
General characteristics	
Sex, female, n (%)	1,126 (50.9)
Birth weight, g, mean ± SD	3460.3 ± 541
Gestational age at birth, wk, mean ± SD	40 ± 1.6
Average child age at assessment, y, mean ± SD	13.5 ± 0.3
Child IQ, mean ± SD	103.8 ± 13.3
Outcome	
CBCL, total behavioral problem score, mean ± SD	18.2 ± 16.3
CBCL, externalizing problem score, mean ± SD	4.2 ± 5.2
CBCL, internalizing problem score, mean ± SD	5.5 ± 5.7

*Note: BMI* = *body mass index; CBCL = Child Behavioral Checklist.*

**TABLE 2:** Standardized Coefficients for Direct Associations of Prenatal Infection With Adolescent Behavior

Outcome	Timing of exposure	β	95% CI	Raw p	Adjusted p
CBCL total behavioral problems	Total pregnancy	0.106	0.065 to 0.148	<.001*	<.001**
	First trimester	0.068	0.027 to 0.110	.001*	.009**
	Second trimester	0.078	0.037 to 0.119	<.001*	.002**
	Third trimester	0.083	0.042 to 0.125	<.001*	.002**
CBCL internalizing behavioral problems	Total pregnancy	0.111	0.069 to 0.152	<.001*	<.001**
	First trimester	0.077	0.035 to 0.118	<.001*	.003**
	Second trimester	0.069	0.027 to 0.110	.001	.009**
	Third trimester	0.093	0.051 to 0.134	<.001*	<.001**
CBCL externalizing behavioral problems	Total pregnancy	0.057	0.015 to 0.099	.007*	.038**
	First trimester	0.030	−0.011 to 0.071	.153	.378
	Second trimester	0.048	0.007 to 0.090	.023*	.094
	Third trimester	0.045	0.004 to 0.087	.031*	.123
Total SRS	Total pregnancy	0.021	−0.020 to 0.062	.320	.623
	First trimester	0.013	−0.028 to 0.054	.535	.843
	Second trimester	0.005	−0.035 to 0.046	.796	.968
	Third trimester	0.027	−0.088 to 0.126	.733	.460

Note: All models were corrected for maternal age, maternal education, paternal education, household income, maternal national background, maternal IQ, child sex, child IQ, and child age at measurement. Adjusted p values are Benjamini–Hochberg corrected. CBCL = Child Behavioral Checklist; SRS = Social Responsiveness Scale.

* p < 0.05;

** p < 0.05 after multiple testing correction.

**TABLE 3:** Standardized Coefficients for Moderating Effects of Moderators

Outcome	Moderator	β	95% CI	Raw *p*	Adjusted *p*
CBCL total behavioral problems	Maternal psychopathology	0.067	0.033 to 0.101	<.001*	.002**
	Maternal dietary food score	0.015	−0.029 to 0.058	.511	.825
	Maternal iron levels	−0.014	−0.056 to 0.028	.516	.825
	Maternal vitamin D levels	−0.038	−0.079 to 0.004	.075	.232
	Pre-pregnancy BMI	−0.017	−0.059 to 0.023	.398	.726
	Maternal substance use (yes)	0.183	0.032 to 0.332	.017*	.075
	Maternal alcohol use (pregnancy)	0.153	0.086 to 0.218	<.001*	<.001**
	Maternal tobacco use (pregnancy)	0.145	0.034 to 0.256	.01*	.050**
	Child BMI	0.007	−0.035 to 0.050	.722	.961
	Child tobacco use (yes)	0.279	−0.852 to 1.411	.622	.906
	Child alcohol use (yes)	0.172	−0.023 to 0.368	.083	.244
	Breastfeeding (no)	0.133	−0.032 to 0.299	.111	.306
	Childhood infections	0.002	−0.074 to 0.078	.951	.984
	Postnatal life events score	0.058	0.019 to 0.096	.003*	.020**
	Postnatal direct victimization score	0.075	0.035 to 0.114	<.001*	.002**
CBCL internalizing behavioral problems	Maternal psychopathology	0.062	0.028 to 0.095	<.001*	.003**
	Maternal dietary food score	0.026	−0.020 to 0.073	.258	.541
	Maternal iron levels	0.004	−0.036 to 0.046	.822	.973
	Maternal vitamin D levels	−0.007	−0.049 to 0.037	.762	.964
	Pre-pregnancy BMI	−0.028	−0.068 to 0.011	.162	.389
	Maternal substance use (yes)	0.215	0.064 to 0.366	.005*	.031**
	Maternal alcohol use (pregnancy)	0.127	0.062 to 0.193	<.001*	.002**
	Maternal tobacco use (pregnancy)	0.183	0.071 to 0.293	.001*	.009**
	Child BMI	−0.007	−0.050 to 0.036	.758	.964
	Child tobacco use (yes)	0.346	−0.711 to 1.404	.516	.825
	Child alcohol use (yes)	0.270	0.068 to 0.472	.009*	.048*
	Breastfeeding (no)	0.126	−0.037 to 0.290	.127	.339
	Childhood infections	−0.007	−0.073 to 0.058	.816	.973
	Postnatal life events score	0.057	0.019 to 0.096	.003*	.021*
	Postnatal direct victimization score	0.074	0.035 to 0.114	<.001*	.002*
CBCL externalizing behavioral problems	Maternal psychopathology	0.024	−0.001 to 0.057	.166	.396
	Maternal dietary food score	0.033	−0.012 to 0.079	.154	.383
	Maternal iron levels	0.005	−0.036 to 0.047	.793	.965
	Maternal vitamin D levels	0.017	−0.026 to 0.061	.429	.761
	Pre-pregnancy BMI	−0.031	−0.072 to 0.009	.133	.353
	Maternal substance use (yes)	0.142	−0.008 to 0.292	.064	.227
	Maternal alcohol use (pregnancy)	0.053	−0.012 to 0.119	.110	.312
	Maternal tobacco use (pregnancy)	0.122	0.011 to 0.233	.031*	.126
	Child BMI	−0.019	−0.062 to 0.024	.391	.738
	Child tobacco use (yes)	0.344	−0.851 to 1.539	.563	.871
	Child alcohol use (yes)	0.231	0.420 to 0.040	.018*	.081
	Breastfeeding (no)	0.056	0.012 to 0.101	.420	.756
	Childhood infections	−0.022	−0.070 to 0.026	.362	.706
	Postnatal life events score	0.032	−0.006 to 0.071	.098	.294
	Postnatal direct victimization score	0.023	−0.016 to 0.062	.253	.548
Total SRS	Maternal psychopathology	0.031	−0.002 to 0.046	.066	.193
	Maternal dietary food score	0.035	−0.013 to 0.083	.147	.486
	Maternal iron levels	0.014	−0.028 to 0.056	.515	.720
	Maternal vitamin D levels	−0.007	−0.048 to 0.034	.735	.777
	Pre-pregnancy BMI	−0.022	−0.063 to 0.020	.306	.609
	Maternal substance use (yes)	0.221	0.073 to 0.369	.003*	.021*
	Maternal alcohol use during pregnancy	0.040	−0.025 to 0.106	.227	.487
	Maternal tobacco use during pregnancy	0.099	−0.011 to 0.210	.077	.235
	Child BMI	0.008	−0.036 to 0.052	.072	.232
	Child tobacco use (yes)	0.307	−0.634 to 1.249	.052	.188
	Child alcohol use (yes)	0.195	0.014 to 0.377	.035*	.135
	Breastfeeding (no)	0.091	−0.054 to 0.237	.219	.486
	Childhood infections	−0.017	−0.064 to 0.030	.474	.798
	Postnatal life events score	0.052	0.013 to 0.090	.008*	.042*
	Postnatal direct victimization score	0.054	0.015 to 0.094	.006*	.035*

Note: All models were corrected for maternal age, maternal education, paternal education, household income, maternal national background, maternal IQ, child sex, child IQ, and child age at measurement. Adjusted p values are Benjamini–Hochberg corrected. BMI = body mass index; CBCL = Child Behavioral Checklist; SRS = Social Responsiveness Scale.

* p < 0.05;

** p < 0.05 after multiple testing correction.

## Diversity & Inclusion Statement:

We worked to ensure race, ethnic, and/or other types of diversity in the recruitment of human participants. We worked to ensure that the study questionnaires were prepared in an inclusive way. We worked to ensure sex and gender balance in the recruitment of human participants.
