# Supplementary material for: The Association Between Prenatal Infection and Adolescent Behavior: Investigating Multiple Prenatal, Perinatal, and Childhood Second Hits
Source: J Am Acad Child Adolesc Psychiatry. Author manuscript; Available in PMC 2024 Mar 30. (PMC10981534; doi:10.1016/j.jaac.2023.06.009)
Supplement: supplementary material [file NIHMS1976832-supplement-supplementary_material.pdf]

SUPPLEMENTARY INFORMATION

The Association Between Prenatal Infection and Adolescent Behavior: Investigating Multiple Prenatal, Perinatal, and Childhood Second Hits

Anna Suleri BSc[1]*, Tonya White MD PhD[1], Elisabet Blok BSc[1], Charlotte Cecil PhD[1], Irwin Reiss MD PhD[1], Vincent W.V. Jaddoe MD PhD[1], Frederieke Gigase MSc[1], Manon Hillegers MD PhD[1], Lot de Witte MD PhD[2], Veerle Bergink MD PhD[1,2†], Anna-Sophie Rommel PhD[2†]

[1]Erasmus University Medical Center, Rotterdam, The Netherlands.

[2]Icahn School of Medicine at Mount Sinai, New York, USA.

[†] Equal contribution

*Corresponding author: a.suleri@erasmusmc.nl

Table of Contents

SUPPLEMENT 1: PREREGISTRATION 2
- PREREGISTRATION DEVIATIONS 2
- PREREGISTRATION RESULTS 2

SUPPLEMENT 2: DISTRIBUTION INFECTION TYPES AND BEHAVIORAL PROBLEMS 10
SUPPLEMENT 3: CORRELATION INFECTION VARIABLES AND CRP 11
SUPPLEMENT 4: SCHEMATIC OVERVIEW METHOD 12
SUPPLEMENT 5: ADDITIONAL INFORMATION: MEDIATING VARIABLES 14
SUPPLEMENT 6: ADDITIONAL INFORMATION: MODERATING VARIABLES 15
SUPPLEMENT 7: FREQUENCY MEDIATORS AND MODERATORS 20
SUPPLEMENT 8: NON-RESPONSE ANALYSIS 23
SUPPLEMENT 9: REGRESSION PLOTS: PRENATAL INFECTION & ADOLESCENT BEHAVIOR 24
SUPPLEMENT 10: MEDIATION RESULTS: UNDERLYING PATHWAY 25
SUPPLEMENT 11: SENSITIVITY ANALYSES RESULTS 29
REFERENCES 36

Supplement 1: Preregistration

Preregistration deviations

As per our preregistration, we attempted to fit structural equation models (SEM). However, the latent variables reported bad fit measures for all modification indices. In line with the alternative strategies outlined in our preregistration, we conducted linear regression analyses to study the effects of prenatal infection on adolescent behavior and performed individual moderation and multiple mediation analyses to investigate the role of various cofactors. There were six deviations. First, the variable maternal age, a factor in one of our preregistered SEM latent variables, was included as an observed covariate in all analyses. Second, due to the large number of individual tests carried out to replace the originally planned SEM, we investigated the effects of infection timing and sex in the linear regression analyses only. Third, we clustered self-reported prenatal infection symptoms into the clinically meaningful categories 'upper respiratory tract infection' (rhinitis, pharyngitis, ear infection and sinusitis), 'lower respiratory tract infection' (pneumonia and bronchitis), and 'gastrointestinal tract infection' (enteritis or diarrhea). Fever as well as other infections, such as cystitis/pyelitis, dermatitis, eye infections, herpes zoster, flu, and sexually transmitted diseases (STD), were treated individually as described in the preregistration. We also investigated 'fever' as a separate severity marker. The results with the preregistered sum score can be found directly below in Tables S1-3. Fourth, we also investigated infection-specific effects on child behavioral problems as a post-hoc analysis. Fifth, we excluded low-frequency mediators (<5%) from the final analyses due to power issues. The following mediators were omitted: gestational diabetes, preeclampsia, and pregnancy-induced hypertension. Sixth, we added the following mediators and moderators based on biological plausibility. Moderators added were 'postnatal life events' and 'postnatal direct victimization'. Mediators added were '5-minute Apgar score' and 'umbilical cord blood pH'.

Preregistration results

Supplementary Tables 1-4 show the results of the preregistered infection sum score (https://osf.io/cp85a) using a False Discovery Rate – Benjamini Hochberg (FDR-BH) correction applied for 104 tests. Similar to the main manuscript, we investigated the direct associations between prenatal infection and adolescent behavior, the moderating effect of perinatal factors and the mediating effects

of various maternal and child factors. The results of the preregistered sum score are similar to the results of the main analysis presented in the manuscript.

Table S1. Direct effects of prenatal maternal infection on adolescent behavior

Outcome	Timing of exposure	ß	95% CI	P-value
CBCL total behavioral problems				
	Total pregnancy	0.120	0.078 – 0.162	<0.001*
	First trimester	0.078	0.035 – 0.119	0.003*
	Second trimester	0.100	0.058 – 0.142	<0.001*
	Third trimester	0.095	0053 – 0.137	<0.001*
CBCL internalizing behavioral problems				
	Total pregnancy	0.122	0.090 – 0.165	<0.001*
	First trimester	0.088	0.046 – 0.131	<0.001*
	Second trimester	0.087	0.045 – 0.129	<0.001*
	Third trimester	0.101	0.059 – 0.144	<0.001*
CBCL externalizing behavioral problems				
	Total pregnancy	0.073	0.030 – 0.115	0.005*
	First trimester	0.032	-0.012 – 0.072	0.404
	Second trimester	0.070	0.028 – 0.112	0.007*
	Third trimester	0.064	0.022 – 0.107	0.003*
Total SRS				
	Total pregnancy	0.023	-0.019 – 0.065	0.547
	First trimester	0.008	-0.034 – 0.050	0.986
	Second trimester	0.014	-0.028 – 0.056	0.837
	Third trimester	0.031	-0.011 – 0.073	0.404
†Benjamini-Hochberg adjusted p-values are reported				

*p < 0.05

Table S2. Moderating effects of prenatal maternal infection on adolescent behavior

Outcome	Moderator	ß	95% CI	P-value
CBCL total behavioral problems				
	Maternal psychopathology	0.070	0.038 – 0.101	<0.001*
	Maternal dietary food score	0.007	-0.039 – 0.054	0.986
	Maternal iron levels	0.030	-0.013 – 0.073	0.404
	Maternal vitamin D levels	0.003	-0.041 – 0.047	0.986
	Pre-pregnancy BMI	-0.023	-0.068 – 0.022	0.567
	Maternal substance use (yes)	0.235	0.065 – 0.405	0.032*
	Maternal alcohol use (pregnancy)	0.151	0.084 – 0.218	<0.001*
	Maternal tobacco use (pregnancy)	0.218	0.104 – 0.333	0.001*
	Child BMI	0.013	-0.033 – 0.059	0.902
	Child tobacco use (yes)	0.003	-1.772 – 1.777	0.998
	Child alcohol use (yes)	0.191	-0.023 – 0.405	0.255

	Breastfeeding (no)	0.209	0.025 – 0.393	0.099
	Childhood infections	0.005	-0.057 – 0.067	0.986
CBCL internalizing behavioral problems				
	Maternal psychopathology	0.072	0.040 – 0.104	<0.001*
	Maternal dietary food score	0.002	-0.043 – 0.046	0.986
	Maternal iron levels	0.012	-0.031 – 0.057	0.902
	Maternal vitamin D levels	-0.026	-0.071 – 0.020	0.558
	Pre-pregnancy BMI	-0.005	-0.051 – 0.041	0.986
	Maternal substance use (yes)	0.187	0.017 – 0.358	0.114
	Maternal alcohol use (pregnancy)	0.153	0.086 – 0.220	<0.001*
	Maternal tobacco use (pregnancy)	0.160	0.045 – 0.275	0.032*
	Child BMI	0.048	0.003 – 0.093	0.526
	Child tobacco use (yes)	0.024	-1.556 – 1.604	0.986
	Child alcohol use (yes)	0.135	-0.058 – 0.328	0.404

	Breastfeeding (no)	0.239	0.063 – 0.416	0.036*
	Childhood infections	0.006	-0.073 – 0.084	0.986
CBCL externalizing behavioral problems				
	Maternal psychopathology	0.027	-0.005 – 0.059	0.290
	Maternal dietary food score	0.020	-0.026 – 0.066	0.699
	Maternal iron levels	0.033	-0.010 – 0.077	0.379
	Maternal vitamin D levels	0.020	-0.023 – 0.064	0.652
	Pre-pregnancy BMI	-0.035	-0.081 – 0.012	0.394
	Maternal substance use (yes)	0.155	-0.016 – 0.326	0.258
	Maternal alcohol use (pregnancy)	0.079	0.012 – 0.146	0.086
	Maternal tobacco use (pregnancy)	0.148	0.033 – 0.264	0.055
	Child BMI	-0.010	-0.055 – 0.035	0.949
	Child tobacco use (yes)	-0.027	-1.935 – 1.881	0.987
	Child alcohol use (yes)	0.149	-0.060 – 0.358	0.389

	Breastfeeding (no)	0.083	0.027 – 0.278	0.699
	Childhood infections	-0.002	-0.064 – 0.059	0.987

†Benjamini-Hochberg adjusted p-values are reported

*p < 0.05

Table S3. Mediating effects of prenatal maternal infection on adolescent behavior

Outcome	Mediator	ß	95% CI	P-value
CBCL total behavioral problems				
	Arteria umbilicalis pulsatility index	0.000	-0.002 – 0.003	0.987
	Arteria uterine resistance index	-0.003	-0.010 – 0.001	0.389
	Placental growth factor	-0.001	-0.007 – 0.002	0.951
	Placental weight	0.000	-0.003 – 0.002	0.987
	Pre-eclampsia	0.001	-0.004 – 0.007	0.987
	Diabetes gravidarum	0.000	-0.002 – 0.005	0.987
	Pregnancy induced hypertension	0.000	-0.002 – 0.003	0.987
	Birthweight	0.000	-0.004 – 0.004	0.987
	Gestational age at birth	-0.001	-0.005 – 0.002	0.840
CBCL internalizing behavioral problems				
	Arteria umbilicalis pulsatility index	0.001	-0.001 – 0.004	0.927
	Arteria uterine resistance index	-0.003	-0.011 – 0.001	0.534
	Placental growth factor	-0.002	-0.009 – 0.001	0.808

	Placental weight	0.000	-0.003 – 0.004	0.987
	Pre-eclampsia	0.001	-0.006 – 0.007	0.987
	Diabetes gravidarum	0.000	-0.002 – 0.003	0.987
	Pregnancy induced hypertension	0.000	-0.003 – 0.003	0.987
	Birthweight	0.000	-0.004 – 0.003	0.987
	Gestational age at birth	-0.001	-0.004 – 0.002	0.951
CBCL externalizing behavioral problems				
	Arteria umbilicalis pulsatility index	0.000	-0.001 – 0.003	0.987
	Arteria uterine resistance index	-0.003	-0.007 – 0.002	0.507
	Placental growth factor	0.000	-0.004 – 0.004	0.840
	Placental weight	0.000	-0.003 – 0.003	0.927
	Pre-eclampsia	0.001	-0.003 – 0.009	0.808
	Diabetes gravidarum	0.001	-0.002 – 0.006	0.987
	Pregnancy induced hypertension	0.000	-0.003 – 0.002	0.987
	Birthweight	0.000	-0.006 – 0.003	0.987
	Gestational age at birth	-0.001	-0.005 – 0.001	0.987
Total SRS				
	Arteria umbilicalis pulsatility index	0.001	-0.002 – 0.004	0.831
	Arteria uterine resistance index	0.000	-0.004 – 0.006	0.986
	Placental growth factor	-0.003	-0.014 – 0.000	0.695
	Placental weight	0.000	-0.002 – 0.000	0.986

	Pre-eclampsia	-0.001	-0.005 – 0.004	0.968
	Diabetes gravidarum	0.000	-0.005 – 0.002	0.968
	Pregnancy induced hypertension	0.000	-0.001 – 0.001	0.968
	Birthweight	0.000	-0.002 – 0.002	0.968
	Gestational age at birth	-0.001	-0.006 – 0.001	0.831

†Benjamini-Hochberg adjusted p-values are reported

*p < 0.05

Supplement 2: Distribution infection types and behavioral problems

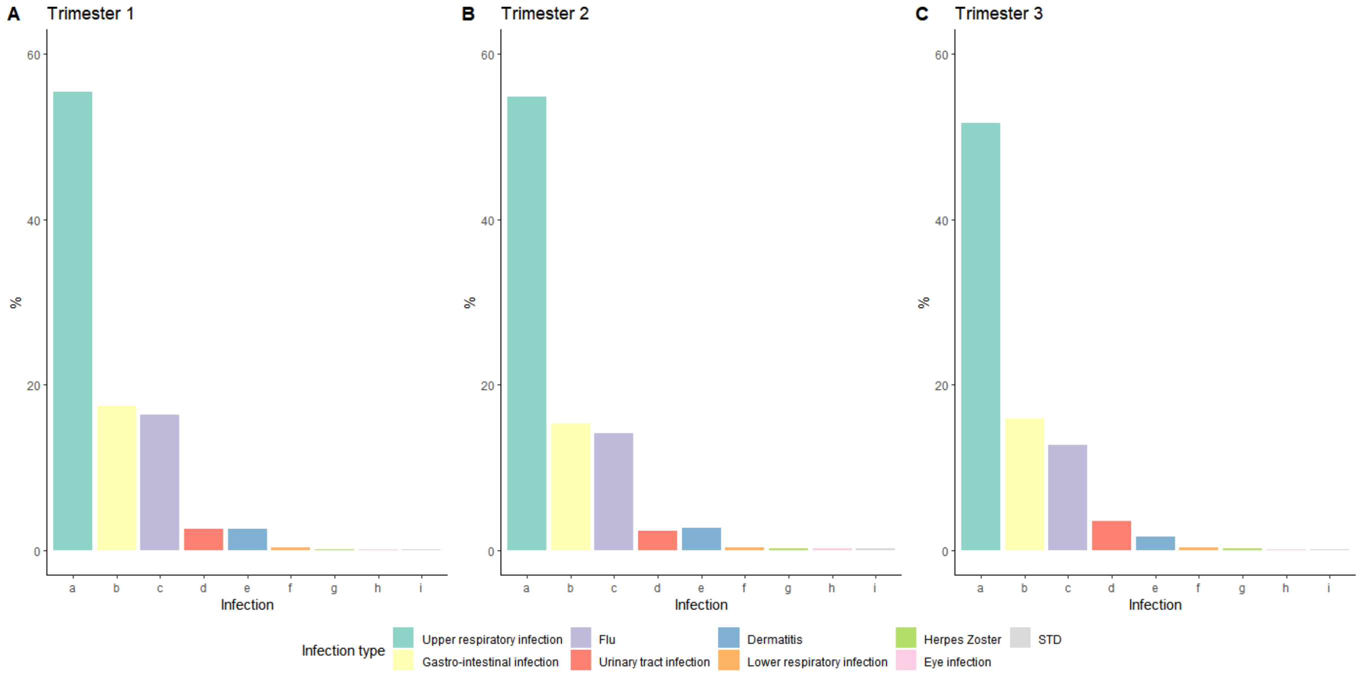

Figure S1. Distribution different infection types. Figures S1A-C show the distribution of the infection for trimesters 1, 2 and 3, respectively.

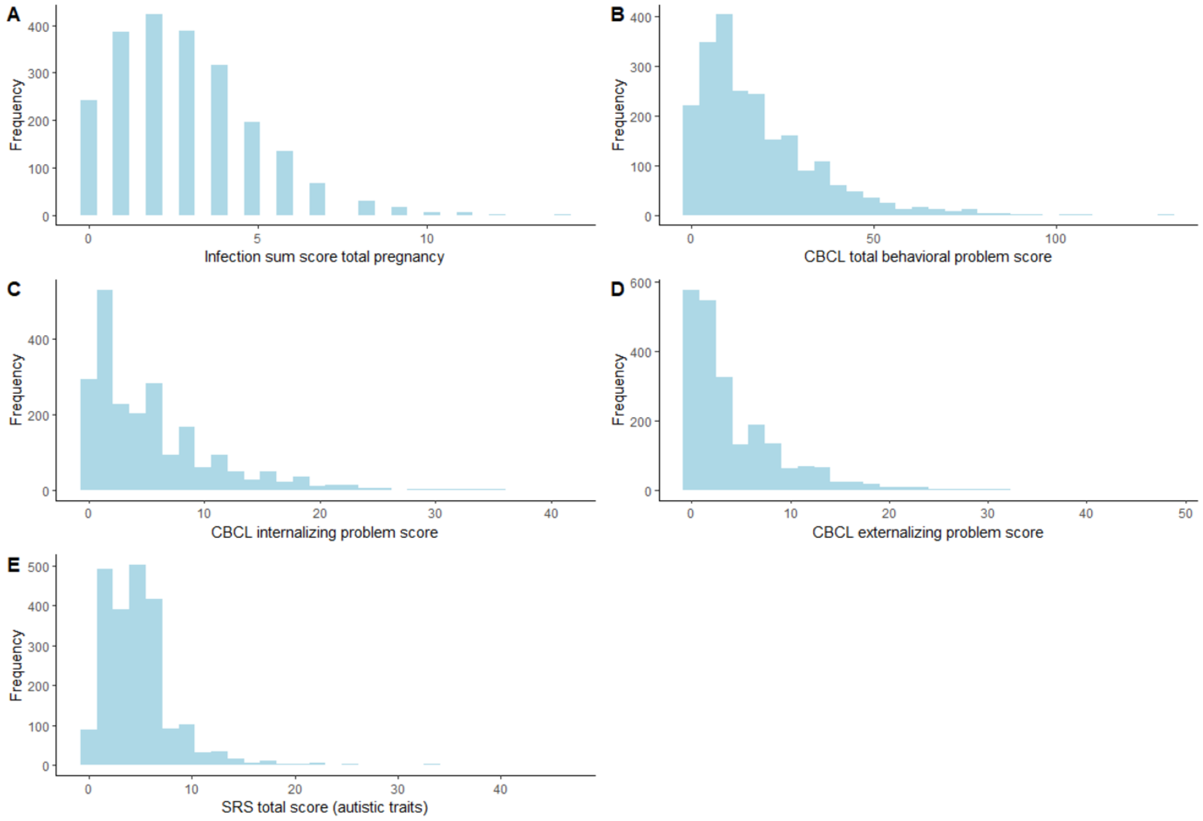

Figure S2. Frequency plot for exposure and outcome variables.

Supplement 3: Correlation infection variables and CRP

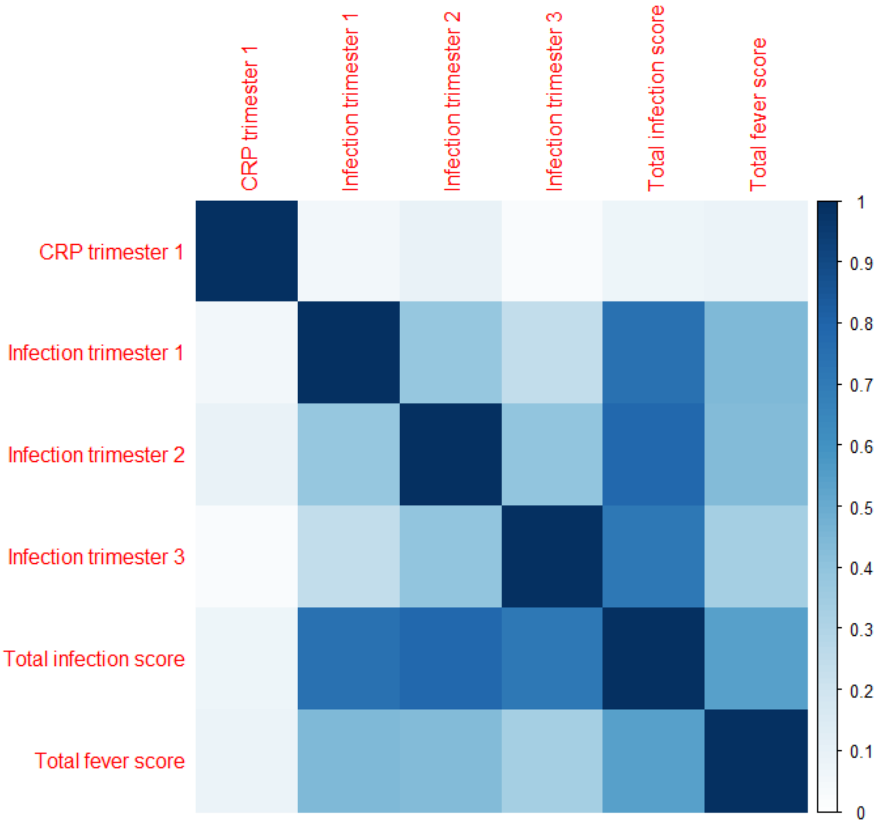

Figure S3. Correlation between C-reactive protein levels <18 weeks of gestation (1 fixed time point measurement available in Generation R) and total and trimester-based prenatal infection scores.

Supplement 4: Schematic overview method

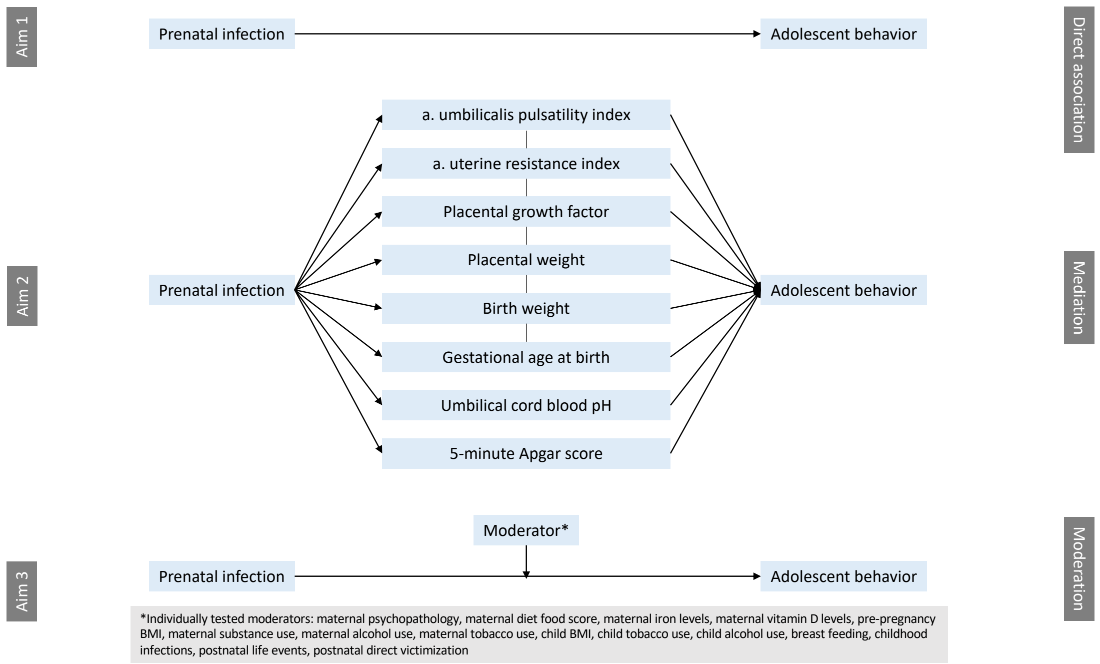

Figure S4. Schematic overview statistical analyses. Mediators were chosen to investigate underlying pathways between prenatal infection and adolescent behavior problems. Specifically, we examined if placental or obstetric variables, previously linked to infection and behavioral problems individually, explained the association investigated in aim 1. Moderators were hypothesized to be downstream stressors ('second hits'), which are not directly associated with prenatal infection but instead are modifiable environmental risk factors affecting the size or direction of the association. Covariates in all analyses are socioeconomic and non-modifiable variables potentially confounding the association for which we wanted to adjust.

**Mediator**[1]	**Moderator**[2]	**Covariate**[3]
a.umbilicalis pulsatility index	Maternal psychopathology	Maternal age
a.uterine resistance index	Maternal diet food score	Maternal education
Placental growth factor	Maternal iron levels	Paternal education
Placental weight	Maternal vitamin D levels	Household income
Birth weight	Pre-pregnancy BMI	Maternal national background
Gestational age at birth	Maternal substance use	Maternal IQ
5-minute Apgar score	Maternal alcohol use	Child IQ
Umbilical cord pH	Maternal tobacco use	Child sex
	Child BMI	Child age
	Child tobacco use	
	Child alcohol use	
	Breast feeding	
	Childhood infections	
	Postnatal life events	

	Postnatal direct victimization	

[1]A **mediator** is a variable that explains the relationship between two other variables. It helps to clarify the mechanism through which the independent variable affects the dependent variable.
[2]A **moderator** is a variable that affects the strength and direction of the relationship between two other variables. It helps to identify under what conditions or for whom the relationship between the independent variable and dependent variable may be stronger or weaker.
[3]A **covariate** is a variable that is controlled or adjusted for, to assess the relationship between two other variables. It helps to account for potential confounding variables that could affect the relationship between the independent variable and dependent variable.

Supplement 5: Additional information: mediating variables

Standardized fetal ultrasound examinations were performed by trained staff in mid pregnancy (18–25 weeks)[17,18] with high intra- and inter-observer reproducibility.[19] More information on pregnancy dating can be found elsewhere.[18,20] To evaluate the uteroplacental and fetoplacental circulation, flow velocity wave forms from the arteria umbilicalis and arteria uterine were recorded in mid pregnancy.[21,22] We used two indirect measures of placental vascular resistance, namely the **umbilical artery pulsatility index** and the **uterine artery mean resistance index**. The umbilical artery pulsatility index was defined as (peak systolic velocity - end diastolic velocity) / divided by mean peak systolic velocity.[22] The uterine artery mean resistance index was defined as: (peak systolic velocity - end diastolic velocity) / by systolic peak velocity.[22] An increase in the uterine artery resistance index and the umbilical artery pulsatility index can be an indicator of increased placental resistance. More information on this measurement can be found elsewhere.[22]

Information on the **placental weight** (in grams) was collected at birth through medical records. **Placental growth factor** (PlGF) was measured in early pregnancy. Details of processing procedures can be found elsewhere.[10,23] In brief, analyses were performed in non-fasting venous blood samples. The Department of Clinical Chemistry of the Erasmus Medical Center analyzed PlGF concentrations using an immunoelectrochemoluminence assay on the Architect System (Abbott Diagnostics B.V., Hoofddorp, the Netherlands).[23]

Gestational age at birth was established via ultrasound examinations during prenatal visits at the research center.[3] Details of the standardized method for fetal ultrasonographic measurements can be found elsewhere.[18] Information on the child's **birthweight** was obtained from midwives and hospital registries.

The 5-minute **Apgar score** was obtained from medical records. It is a quick assessment tool to evaluate the physical condition of the newborn baby based on the following five components (each of which is scored on a scale of 0-2): heart rate, respiratory effort, muscle tone, reflex irritability, and skin color.

The maximum score is 10. A score of 7 or above is generally considered normal, while a score of 4-6 is considered moderately abnormal, and a score of 0-3 is considered severely abnormal.

Samples of cord blood treated with heparin were collected from an **umbilical cord** segment that was immediately clamped and isolated. The cord blood gases were assessed using automated gas check machines, which analyzed **pH**, pO2, and pCO2, and calculated base excess. The interval between the collection of cord blood samples and their analysis was under 60 minutes. The blood gas analyzers were calibrated and standardized utilizing pH solutions and reference gases.

Supplement 6: Additional information: moderating variables

The **level of vitamin D** was assessed by measuring 25-hydroxyvitamin D in the unit nmol/L in the mother's serum during the first eighteen weeks of gestation.[7] 25-hydroxyvitamin D was defined as the sum of 25-hydroxyvitamin D2 (25OHD2) and 25-hydroxyvitamin D3 (25OHD3).[8] We will use this variable as a continuous variable. To quantify the samples, isotope dilution liquid chromatography–tandem mass spectrometry was used. The analytical system consisted of a Shimadzu Nexera UPLC coupled to an AbSciex 5500 QTRAP equipped with an atmospheric-pressure chemical ionisation source. To evaluate the accuracy of the assay, we used certified reference materials, which were purchased from the National Institute of Standards and Technology (NIST SRM 972a Levels 1–4). From July 2013 to August 2014, samples were analyzed at the Queensland Brain Institute in Brisbane, Australia. Further information of the assay methodology have been described elsewhere.[9] Maternal blood serum samples were collected during early pregnancy. More information about the transportation and storage method can be found elsewhere.[10]

We used iron biomarker **ferritin** as a measure of iron store.[11] Ferritin was determined by electrochemiluminescence immunoassay on the Cobas e411 analyzer (Roche) in the unit micrograms/L.

To evaluate the **mother's diet** in early pregnancy, a semi-quantitative 293-item food frequency questionnaire (FFQ) was used at enrollment. The FFQ comprises frequently consumed Dutch food and was modified for use during pregnancy.[2] Calculation of the energy and nutrient intakes was based on the 2006 Dutch food composition table.[12] Three 24-hour recalls of 71 pregnant women, who lived in Rotterdam, were used to validate the FFQ.[13] With the national dietary guidelines[14], a predefined diet quality score was constructed for pregnant women. The next 15 components and corresponding cut-offs were included for the diet quality score: vegetables (≥200 grams/day), fruit (≥200 grams/ day), whole grains (≥90 grams/ day), legumes (≥135 grams/week), nuts (≥15 grams/day), dairy (≥300 grams/ day), fish (≥100 grams/week), tea (≥450 grams/ day), grain quality (ratio whole grains of total grains), soft fats and oils (ratio of total fat), red meat (≤375 grams/week), sugar-containing beverages (≤150 grams/ day), alcohol (yes/no), salt (≤6 grams/ day), and folic acid supplements in early pregnancy (periconceptional/first ten weeks/not). The scores for all the individual components were summed up, which consequently led to an overall continuous score with a range from 0 to 15. A higher score indicates a healthier diet. More information on the construction of the score can be found elsewhere.[15]

The **pre-pregnancy body mass index (BMI)** was assessed by self-report using a questionnaire at enrollment. Mothers were asked to report their pre-pregnancy weight in kilograms and their height in centimeters, which were used to calculated BMI in kg/m^2. Previous studies in Generation R have shown the correlation between self-reported pre-pregnancy weight and weight measured at enrollment to be 0.97 ($P < 0.01$).[16]

Information on **maternal psychoactive substance use during pregnancy** was assessed with a questionnaire at enrollment. Maternal psychoactive substance usage (i.e., marihuana, hashish, cocaine, heroin, or ecstasy) was categorized into: 'no', 'yes, until pregnancy was known,' and 'yes, continued during pregnancy.' Information on **maternal tobacco use during pregnancy** was assessed with a questionnaire at enrollment. Maternal tobacco use was categorized into: 'no', 'yes, until pregnancy was known,' and 'yes, continued during pregnancy.' Information on **maternal alcohol consumption during pregnancy** was assessed using a questionnaire at enrollment. Maternal alcohol consumption encompassed the categories: 'none during pregnancy,' 'drank until pregnancy was

known,' 'continued drinking occasionally,' and 'continued drinking frequently' (one or more glass/week for at least two trimesters).

Prenatal maternal psychopathology was measured with a validated self-reported questionnaire (Brief Symptom Inventory) at enrollment. From this 53-item questionnaire[1], a Global Severity Index was calculated that served as a continuous score of prenatal maternal psychopathology with higher scores indicating more problems.

Information about **breastfeeding** was obtained from delivery reports and postnatal questionnaires at the ages of 2, 6 and 12 months after birth.[4] Mothers were asked whether they ever breastfed their child, with the answer possibilities were: 'yes' or 'no'. In this study, breastfeeding was included as a binary variable (ever/never).

Information on **childhood body mass index (BMI)** was collected when the children visited the research center at 9-12 years of age. The height and weight of the child were measured by trained staff. Height was measured with a stadiometer (Holtain Limited) and weight was measured with an electronical scale (SECA).[24] When measuring weight, any heavy clothing or shoes were taken off. BMI (kg/m^2) was calculated using height and weight. We used a measure of BMI which is adjusted for age and sex based on Dutch growth curves[25], also called BMI-SDS. BMI-SDS is an index of the nutritional status of the child.[26] More information can be found elsewhere.[24]

Information about the **child's substance use (alcohol and tobacco)** was obtained using a self-report questionnaire at age 13-16 years. The children were asked if they ever drank alcohol and if they had ever smoked tobacco. Both variables were coded as 'yes', 'no', and 'don't know'.

A sum score for **childhood infection** was created based on information at seven different time points. In Table S5, the time point, the question, the answer possibilities, and the scoring can be found. A total of 15 points could be scored, with a higher score representing a higher number of infections.

Table S4. Calculation of sum score for childhood infection

Timepoint	Question	Answer possibilities	Scoring
2 months	Has your child ever used one or more of the following medicines (antibiotics/penicillin)?	Never	0
		Yes, for 1 period of sickness	1
		Yes, for 2 or more periods of sickness	2
6 months	Has your child ever used one or more of the following medicines (antibiotics/penicillin)?	Never	0
		Yes, for 1 bout of illness	1
		Yes, for 2 or more bouts of illness	2
1 years	Has your child used one or more of the following medicines (antibiotics/penicillin) in the last 6 months?	Never	0
		Yes, for 1-2 sickness periods	1.5
		Yes, for 3-4 sickness periods	3.5
		Yes, for more than 5 sickness periods	5
2 years	Has your child been given one or more of the following medicines (antibiotics or penicillin) during the past year?	Never	0
		Yes, 1-2 periods of illness	1.5
		Yes, 3-4 periods of illness	3.5
		Yes, 5 or more periods of illness	5
3 years		Never	0

	Has your child been given one or more of the following medicines (antibiotics or penicillin) during the past year?	Yes, 1-2 periods of illness	1.5
		Yes, 3-4 periods of illness	3.5
		Yes, 5 or more periods of illness	5
4 years	Has your child been given one or more of the following medicines (antibiotics or penicillin) during the past year?	Never	0
		Yes, 1-2 periods of sickness	1.5
		Yes, 3-4 periods of sickness	3.5
		Yes, 5 or more periods of sickness	5
5 years	Was your child given antibiotics (for example, penicillin) during the past year because of a fever?	Never	0
		Yes, 1-2 periods of illness	1.5
		Yes, 3-4 periods of illness	3.5
		Yes, 5 or more periods of illness	5
9 years	In the past year did your child use any antibiotics (for example penicillin) for fever or infection?	Never	0
		Yes, once or twice	1.5
		Yes, 3 times or more	3

Supplement 7: Frequency mediators and moderators

Table S5. Frequency table of all mediators and moderators

**Maternal variables**	
Age at enrolment (mean, SD)	31.2 ± 4.3
Pre-pregnancy BMI (mean, SD)	23.3 ± 4.0
Maternal psychopathology (GSI) (median, IQR)	0.4 (0.2)
Diet quality food score (mean, SD)	8 ± 1.5
Iron (µg/L) (median, IQR)	60.1 (60.1)
Vitamin D (nmol/L) (mean, SD)	63.3 ± 30
Alcohol consumption (N, %)	
- Never drank in pregnancy	741 (33.5)
- Drank until pregnancy was known	352 (15.9)
- Continued drinking occasionally	857 (38.7)
- Continued drinking frequently (>1 glass/week for at least 2 trimesters)	253 (11.4)
- Missing	10 (0.5)
Tobacco use (N, %)	
- Never smoked during pregnancy	1720 (77.7)
- Smoked until pregnancy was known	207 (9.4)
- Continued smoking in pregnancy	264 (11.9)
- Missing	22 (1.0)
Substance use (N, %)	
- Yes	131 (5.9)
- No	2070 (93.5)
- Missing	12 (0.5)
Arteria umbilicalis (pulsatility index) (mean, SD)	1.2 ± 0.2
Arteria uterine (resistance index) (mean, SD)	0.5 ± 0.1

Placental weight (g) (mean, SD)	635.9 ± 146.5
PIGF (pg/ml) (median, IQR)	38.3 (34.4)
Pregnancy induced hypertension (N, %)	
- Yes	97 (4.4)
- No	2070 (93.5)
- Missing	46 (2.1)
Preeclampsia (N, %)	
- Yes	36 (1.6)
- No	2114 (95.5)
- Missing	63 (2.8)
Diabetes gravidarum (N, %)	
- Yes	23 (3.2)
- No	2119 (95.8)
- Missing	71 (1.0)
**Child variables**	
Birth weight (g) (mean, SD)	3460.3 ± 541
Gestational age at birth (weeks) (mean, SD)	40 ± 1.6
5-minute Apgar score (mean, SD)	9.6 ± 0.8
Umbilical cord blood pH (mean, SD)	7.3 ± 0.1
BMI-SDS (mean, SD)	0.2 ± 1.0
Alcohol consumption (N, %)	
- Yes	85 (3.8)
- No	1626 (73.5)
- Do not know	45 (2.0)
- Missing	457 (20.7)
Tobacco use (N, %)	
- Yes	4 (0.2)

- No	1743 (78.8)
- Do not know	4 (0.2)
- Missing	462 (20.9)
Breastfed (N, %)	
- Yes	1797 (81.2)
- No	140 (6.3)
- Missing	276 (12.5)
Childhood infection sum score (median, IQR)	2.5 (3.5)
Postnatal life events score (median, IQR)	0.1 (0.1)
Postnatal direct victimization score (median, IQR)	0.2 (0.2)
†Mean ± SD are noted for normally distributed variables and median (IQR) are noted for skewed variables.	

Supplement 8: Non-response analysis

A comparison between included and excluded mothers revealed that included mothers were older (mean difference=1.7 years, df=4310, p<0.001). Moreover, included mothers more often had a Dutch national background (χ^2=446, df=1, p<0.001), higher educational levels (χ^2=209.3, df=2, p<0.001) and household income (χ^2=7.2, df=1, p=0.007). Fathers of the included children also had higher educational levels (χ^2=153.1, df=2, p<0.001). Included children had higher IQs (mean difference=3.45 points, df=4214.5, p<0.001).

Supplement 9: Regression plots: prenatal infection & adolescent behavior

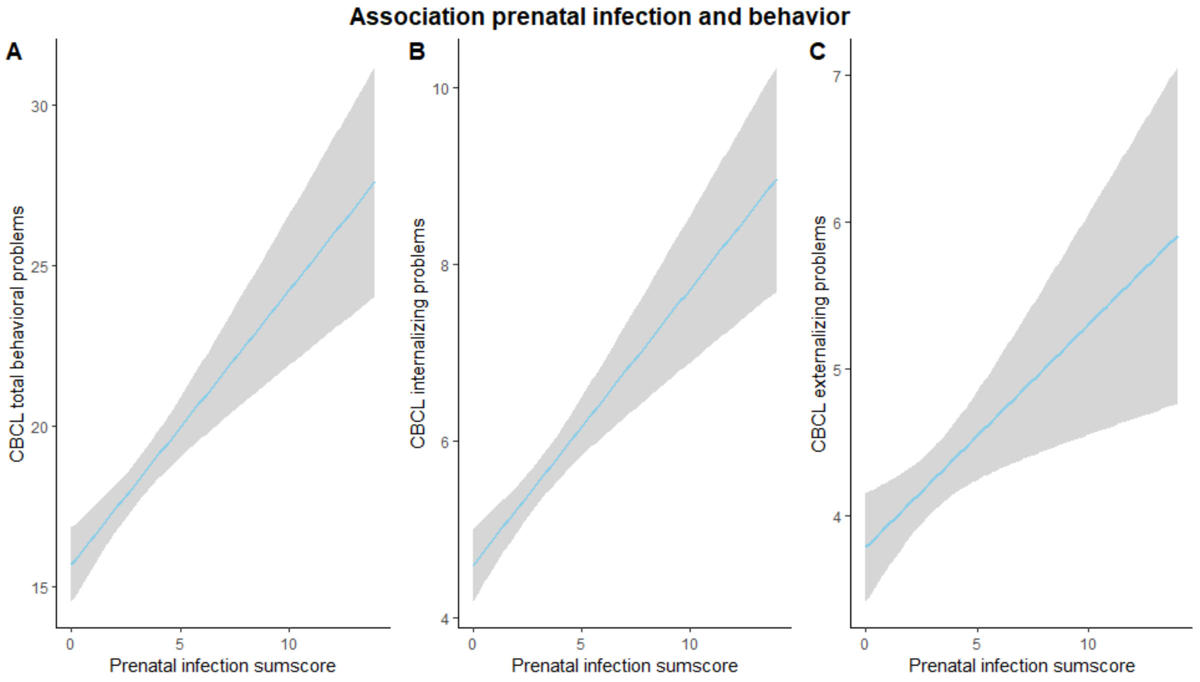

Figure S5. Association between prenatal infection and adolescent behavior. Figures S4A-C visualize the linear regression between prenatal infection and for each adolescent behavioral problems outcome (total behavioral problems, internalizing problems, externalizing problems), respectively.

Supplement 10: Mediation results: underlying pathway

Table S6. Standardized coefficients for indirect effects of mediators.

Outcome	Mediator	ß	95% CI	Raw p-value	Adjusted p-value
CBCL total behavioral problems					
	Arteria umbilicalis pulsatility index	0.000	-0.002 – 0.002	0.948	0.984
	Arteria uterine resistance index	-0.002	-0.006 – 0.001	0.211	0.303
	Placental growth factor	0.000	-0.005 – 0.001	0.901	0.909
	Placental weight	0.000	-0.005 – 0.003	0.800	0.984
	Birthweight	0.001	-0.002 – 0.006	0.456	0.476
	Gestational age at birth	-0.001	-0.006 – 0.001	0.905	0.984
	5-minute Apgar score	0.000	-0.001 – 0.003	0.760	0.988
	Umbilical cord blood pH	0.000	-0.006 – 0004	0.975	0.988
CBCL internalizing behavioral problems					

	Arteria umbilicalis pulsatility index	0.000	-0.003 – 0.002	0.576	0.783
	Arteria uterine resistance index	-0.001	-0.006 – 0.002	0.666	0.751
	Placental growth factor	0.000	-0.004 – 0.002	0.838	0.984
	Placental weight	-0.001	-0.009 – 0.002	0.591	0.753
	Birthweight	0.001	-0.002 – 0.004	0.863	0.973
	Gestational age at birth	0.000	-0.005 – 0.005	0.897	0.984
	5-minute Apgar score	0.000	-0.004 – 0.002	0.833	0.988
	Umbilical cord blood pH	0.000	-0.004 – 0.005	0.972	0.988
CBCL externalizing behavioral problems					
	Arteria umbilicalis pulsatility index	0.000	-0.002 – 0.003	0.735	0.973
	Arteria uterine resistance index	-0.002	-0.008 – 0.003	0.778	0.973
	Placental growth factor	0.000	-0.003 – 0.002	0.443	0.615
	Placental weight	0.000	-0.006 – 0.005	0.680	0.783

	Birthweight	0.001	-0.003 – 0.006	0.610	0.751
	Gestational age at birth	-0.001	-0.006 – 0.003	0.566	0.615
	5-minute Apgar score	0.000	-0.003 – 0.002	0.829	0.988
	Umbilical cord blood pH	0.000	-0.005 – 0.005	0.974	0.988
Total SRS					
	Arteria umbilicalis pulsatility index	0.000	-0.004 – 0.003	0.731	0.973
	Arteria uterine resistance index	0.001	-0.004 – 0.006	0.389	0.599
	Placental growth factor	-0.001	-0.006 – 0.002	0.999	0.999
	Placental weight	0.001	-0.002 – 0.007	0.850	0.973
	Birthweight	0.000	-0.007 – 0.001	0.873	0.973
	Gestational age at birth	-0.002	-0.040 – 0.054	0.973	0.984
	5-minute Apgar score	0.001	-0.001 - 0.003	0.528	0.988
	Umbilical cord blood pH	0.000	-0.004 – 0.006	0.988	0.988
	†All models were corrected for maternal age, maternal education, paternal education, household income, maternal national background, maternal IQ, child sex, child IQ, and child age at measurement.				

	†Adjusted p-values are Benjamini-Hochberg adjusted<br><br>*$p < 0.05$<br><br>**$p < 0.05$ after multiple testing correction

Supplement 11: Sensitivity analyses results

Table S7. Standardized coefficients for direct associations of fever on adolescent behavior

Outcome	Timing of exposure	ß	95% CI	P-value
CBCL total behavioral problems				
	Total pregnancy	0.048	0.006 – 0.099	0.024*
	First trimester	0.049	0.008 – 0.090	0.020
	Second trimester	0.020	-0.021 – 0.061	0.328
	Third trimester	0.028	-0.041 – 0.069	0.190
CBCL internalizing behavioral problems				
	Total pregnancy	0.034	-0.008 – 0.076	0.112
	First trimester	0.043	0.001 – 0.084	0.044*
	Second trimester	0.001	-0.041 – 0.042	0.969
	Third trimester	0.025	-0.016 – 0.067	0.235
CBCL externalizing behavioral problems				
	Total pregnancy	0.021	-0.021 – 0.063	0.328
	First trimester	0.025	-0.016 – 0.067	0.234
	Second trimester	0.009	-0.032 – 0.050	0.651
	Third trimester	0.008	-0.033 – 0.050	0.703
Total SRS				
	Total pregnancy	0.027	-0.014 – 0.068	0.196
	First trimester	0.034	-0.007 – 0.076	0.101
	Second trimester	0.006	-0.035 – 0.048	0.754

	Third trimester	0.011	-0.030 – 0.052	0.592
	†All models were corrected for maternal age, maternal education, paternal education, household income, maternal national background, maternal IQ, child sex, child IQ, and child age at measurement.<br>†Adjusted p-values are Benjamini-Hochberg adjusted<br>*p < 0.05<br>**p < 0.05 after multiple testing correction			

Table S8. Testing interaction term for sex (exposure: prenatal infection)

Outcome	ß	95% CI	P-value
CBCL total behavioral problems	0.020	-0.063 – 0.103	0.633
CBCL internalizing behavioral problems	0.032	-0.051 – 0.114	0.450
CBCL externalizing behavioral problems	0.025	-0.057 – 0.109	0.541
Total SRS	-0.033	-0.116 – 0.049	0.422

Table S9. Testing different infection types and adolescent behavior

Outcome	Infection type	ß	95% CI	P-value
CBCL total behavioral problems				
	Upper respiratory infection	0.084	0.042 – 0.125	<0.001*
	Lower respiratory infection	-0.028	-0.069 – 0.012	0.178
	Gastrointestinal tract infection	0.074	0.033 – 0.115	<0.001*
	Cystitis	0.036	-0.005 – 0.077	0.089
	Dermatitis	0.023	-0.018 – 0.064	0.268
	Eye infections	0.003	-0.037 – 0.045	0.865
	Flu	0.043	0.001 – 0.085	0.040*
	Sexual transmitted disease	0.007	-0.034 – 0.048	0.738
	Herpes zoster	-0.012	-0.054 – 0.028	0.545
CBCL internalizing behavioral problems				
	Upper respiratory infection	0.095	0.053 – 0.136	<0.001*
	Lower respiratory infection	-0.055	-0.096 – -0.013	0.009*

	Gastrointestinal tract infection	0.068	0.026 – 0.109	0.001*
	Cystitis	0.039	-0.002 – 0.081	0.066
	Dermatitis	0.043	0.001 – 0.084	0.044*
	Eye infections	-0.009	-0.050 – 0.032	0.676
	Flu	0.053	0.010 – 0.095	0.014*
	Sexual transmitted disease	0.018	-0.023 – 0.059	0.399
	Herpes zoster	-0.016	-0.057 – 0.025	0.447
CBCL externalizing behavioral problems				
	Upper respiratory infection	0.040	-0.001 – 0.082	0.056
	Lower respiratory infection	-0.017	-0.058 – 0.024	0.414
	Gastrointestinal tract infection	0.063	0.021 – 0.104	0.002*
	Cystitis	0.023	-0.018 – 0.065	0.271
	Dermatitis	0.001	-0.040 – 0.065	0.975
	Eye infections	0.002	-0.039 – 0.042	0.914

	Flu	0.012	-0.029 – 0.044	0.559
	Sexual transmitted disease	-0.005	-0.046 – 0.036	0.797
	Herpes zoster	-0.009	-0.050 – 0.032	0.665
Total SRS				
	Upper respiratory infection	-0.016	-0.057 – 0.025	0.445
	Lower respiratory infection	-0.024	-0.065 – 0.017	0.247
	Gastrointestinal tract infection	0.025	-0.015 – 0.067	0.223
	Cystitis	0.028	-0.013 – 0.069	0.185
	Dermatitis	0.010	-0.030 – 0.052	0.613
	Eye infections	0.007	-0.033 – 0.049	0.716
	Flu	0.026	-0.015 – 0.068	0.218
	Sexual transmitted disease	-0.035	-0.076 – 0.006	0.094
	Herpes zoster	0.002	-0.039 – 0.043	0.919

†All models were corrected for maternal age, maternal education, paternal education, household income, maternal national background, maternal IQ, child sex, child IQ, and child age at measurement.

*$p < 0.05$

References

1. de Beurs E. Brief Symptom Inventory, handleiding. Published online 2004.

2. Klipstein-Grobusch K, den Breeijen JH, Goldbohm RA, et al. Dietary assessment in the elderly: validation of a semiquantitative food frequency questionnaire. *Eur J Clin Nutr*. 1998;52(8):588-596. doi:10.1038/sj.ejcn.1600611

3. El Marroun H, Zou R, Leeuwenburg MF, et al. Association of Gestational Age at Birth With Brain Morphometry. *JAMA Pediatr*. 2020;174(12):1149. doi:10.1001/jamapediatrics.2020.2991

4. Jaddoe VWV, van Duijn CM, van der Heijden AJ, et al. The Generation R Study: design and cohort update 2010. *Eur J Epidemiol*. 2010;25(11):823-841. doi:10.1007/s10654-010-9516-7

5. Schuurmans IK, Luik AI, de Maat DA, Hillegers MHJ, Ikram MA, Cecil CAM. The association of early life stress with IQ-achievement discrepancy in children: A population-based study. *Child Dev*. Published online July 13, 2022. doi:10.1111/cdev.13825

6. Defina S. Cumulative-ELS-score. https://github.com/SereDef/cumulative-ELS-score

7. Institute of Medicine. Dietary Reference Intakes for Calcium and Vitamin D. *National Academies Press*. Published online 2010.

8. Eyles D, Anderson C, Ko P, et al. A sensitive LC/MS/MS assay of 25OH vitamin D3 and 25OH vitamin D2 in dried blood spots. *Clin Chim Acta Int J Clin Chem*. 2009;403(1-2):145-151. doi:10.1016/j.cca.2009.02.005

9. Vinkhuyzen AAE, Eyles DW, Burne TH, et al. Prevalence and predictors of vitamin D deficiency based on maternal mid-gestation and neonatal cord bloods: The Generation R Study. *J Steroid Biochem Mol Biol*. 2016;164:161-167. doi:10.1016/j.jsbmb.2015.09.018

10. Kruithof CJ, Kooijman MN, van Duijn CM, et al. The Generation R Study: Biobank update 2015. *Eur J Epidemiol*. 2014;29(12):911-927. doi:10.1007/s10654-014-9980-6

11. Quezada-Pinedo HG, Mensink-Bout SM, Reiss IK, Jaddoe VWV, Vermeulen MJ, Duijts L. Maternal iron status during early pregnancy and school-age, lung function, asthma, and allergy: The Generation R Study. *Pediatr Pulmonol*. 2021;56(6):1771-1778. doi:10.1002/ppul.25324

12. Netherlands Nutrition Center. *Dutch Food Composition Database 2006 (NEVO 2006)*. The Hague, The Netherlands: Netherlands Nutrition Center.; 2006.

13. Tielemans MJ, Erler NS, Leermakers ETM, et al. A Priori and a Posteriori Dietary Patterns during Pregnancy and Gestational Weight Gain: The Generation R Study. *Nutrients*. 2015;7(11):9383-9399. doi:10.3390/nu7115476

14. Health Council of The Netherlands. Guidelines for a healthy diet 2015. Published online 2015.

15. Nguyen AN, de Barse LM, Tiemeier H, et al. Maternal history of eating disorders: Diet quality during pregnancy and infant feeding. *Appetite*. 2017;109:108-114. doi:10.1016/j.appet.2016.11.030

16. Micali N, De Stavola B, dos-Santos-Silva I, et al. Perinatal outcomes and gestational weight gain in women with eating disorders: a population-based cohort study. *BJOG Int J Obstet Gynaecol*. 2012;119(12):1493-1502. doi:10.1111/j.1471-0528.2012.03467.x

17. Kooijman MN, Kruithof CJ, van Duijn CM, et al. The Generation R Study: design and cohort update 2017. *Eur J Epidemiol*. 2016;31(12):1243-1264. doi:10.1007/s10654-016-0224-9

18. Verburg BO, Steegers EAP, De Ridder M, et al. New charts for ultrasound dating of pregnancy and assessment of fetal growth: longitudinal data from a population-based cohort study. *Ultrasound Obstet Gynecol*. 2008;31(4):388-396. doi:10.1002/uog.5225

19. Verburg BO, Mulder PGH, Hofman A, Jaddoe VWV, Witteman JCM, Steegers EAP. Intra- and interobserver reproducibility study of early fetal growth parameters. *Prenat Diagn*. 2008;28(4):323-331. doi:10.1002/pd.1972

20. Mook-Kanamori DO, Steegers EAP, Eilers PH, Raat H, Hofman A, Jaddoe VWV. Risk factors and outcomes associated with first-trimester fetal growth restriction. *JAMA*. 2010;303(6):527-534. doi:10.1001/jama.2010.78

21. Verburg BO, Jaddoe VWV, Wladimiroff JW, Hofman A, Witteman JCM, Steegers EAP. Fetal hemodynamic adaptive changes related to intrauterine growth: the Generation R Study. *Circulation*. 2008;117(5):649-659. doi:10.1161/CIRCULATIONAHA.107.709717

22. Gaillard R, Arends LR, Steegers EAP, Hofman A, Jaddoe VWV. Second- and third-trimester placental hemodynamics and the risks of pregnancy complications: the Generation R Study. *Am J Epidemiol*. 2013;177(8):743-754. doi:10.1093/aje/kws296

23. Coolman M, Timmermans S, de Groot CJM, et al. Angiogenic and fibrinolytic factors in blood during the first half of pregnancy and adverse pregnancy outcomes. *Obstet Gynecol*. 2012;119(6):1190-1200. doi:10.1097/AOG.0b013e318256187f

24. Jansen PW, Roza SJ, Jaddoe VW, et al. Children's eating behavior, feeding practices of parents and weight problems in early childhood: results from the population-based Generation R Study. *Int J Behav Nutr Phys Act*. 2012;9:130. doi:10.1186/1479-5868-9-130

25. Fredriks AM, van Buuren S, Burgmeijer RJ, et al. Continuing positive secular growth change in The Netherlands 1955-1997. *Pediatr Res*. 2000;47(3):316-323. doi:10.1203/00006450-200003000-00006

26. Zannolli R, Morgese G. Distribution of BMI in children: prevalence of wasting and fattening conditions. *Ann Hum Biol*. 1996;23(1):63-69. doi:10.1080/03014469600004272

27. Raven J. Advanced progressive matrices: Sets Iand II. London. *UK: HK Lewis*. Published online 1962.

28. Chiesi F, Ciancaleoni M, Galli S, Primi C. Using the Advanced Progressive Matrices (Set I) to assess fluid ability in a short time frame: an item response theory-based analysis. *Psychol Assess*. 2012;24(4):892-900. doi:10.1037/a0027830

29. Kaufman A, Raiford S, Coalson D. Intelligent testing with the WISC-V. *John Wiley & Sons*. Published online 2015.

30. Blok E, Schuurmans I, Tijburg A, et al. Cognitive performance in children and adolescents with psychopathology traits: A cross-sectional multicohort study in the general population. *Development and Psychopathology*. Published online 2022:1-15. doi:10.1017/S0954579422000165

31. Netherlands Bureau for Economic Policy Analysis. updated May 9, 2017. http://www.cpb.nl.

32. Schaart R, Moens M, Westerman S. The Dutch Standard Classification of Education, SOI 2006.
